# Anterior Lamina Cribrosa Insertion in Primary Open-Angle Glaucoma Patients and Healthy Subjects

**DOI:** 10.1371/journal.pone.0114935

**Published:** 2014-12-22

**Authors:** Kyoung Min Lee, Tae-Woo Kim, Robert N. Weinreb, Eun Ji Lee, Michaël J. A. Girard, Jean Martial Mari

**Affiliations:** 1 Department of Ophthalmology, Seoul National University College of Medicine, Seoul, Korea; 2 Department of Ophthalmology and Hamilton Glaucoma Center, University of California San Diego, La Jolla, California, United States of America; 3 Department of Bioengineering, National University of Singapore, Singapore, Singapore; 4 Singapore Eye Research Institute, Singapore, Singapore; 5 INSERM U1032, Université de Lyon, Lyon, France; University of Melbourne, Australia

## Abstract

**Purpose:**

To determine using swept-source optical coherence tomography (SS-OCT) whether there are differences in the location of the anterior lamina cribrosa insertion (ALI) in primary open-angle glaucoma (POAG) patients and healthy subjects.

**Methods:**

Fifty three eyes from 53 patients with POAG, and 53 eyes from 53 age-matched healthy subjects were included prospectively in Seoul National University Bundang Hospital. Twelve radial line B-scans centered on the optic disc in every half-clock-hour meridian were acquired using SS-OCT. The ALI position was assessed by measuring two parameters: (1) ALI distance (ALID)—the distance from the anterior scleral canal opening (ASCO) to the ALI; and (2) marginal anterior lamina cribrosa surface depth (mALCSD)—the perpendicular distance from the ASCO plane to the anterior lamina cribrosa surface. These parameters were compared between the two groups for each meridian.

**Results:**

Both ALID (256±54 vs. 209±37 µm, mean ± SD, *p*<0.001) and mALCSD (232±63 vs. 187±40 µm, *p*<0.001) were significantly greater in the POAG group than in the normal group. The largest difference was observed at the 6.5 o′clock and 11.5 o′clock meridians for both ALID and mALCSD. Multiple regression analysis revealed a negative correlation between age and both ALID and mALCSD in the control group, and a negative correlation between mean deviation of the visual field test and both ALID and mALCSD in the POAG group.

**Conclusions:**

The ALI was displaced posteriorly in eyes with POAG compared to those of healthy controls. This finding suggests that the posteriorly located lamina cribrosa insertion is an important component of glaucomatous optic nerve excavation.

## Introduction

Glaucoma is characterized by the loss of retinal ganglion cells and their axons, which is accompanied by corresponding visual field defects [1]. It is generally considered that glaucomatous axonal damage occurs principally at the lamina cribrosa [2]–[4]. Compression and displacement of the lamina cribrosa are thought to contribute to or initiate the blockade of axoplasmic flow within the retinal ganglion cell axons that ultimately leads to the death of retinal ganglion cells [4]. Structural alteration of the lamina cribrosa and the resulting pre-laminar tissue loss cause cupping (excavation) of the optic nerve head. Strain within the lamina cribrosa also could compress the laminar capillaries, causing ischemic insult to the axons [5], [6]. Active cell mediated remodeling of the extracellular matrix [7], [8] has also been suggested as an important part/mechanism of glaucomatous excavation [9].

Yang et al. recently demonstrated the occurrence of posterior migration of the laminar insertion in a primate glaucoma model in which cupping was detected by confocal scanning laser tomography after exposure to moderate intraocular pressure (IOP) elevations [10]. This finding suggests that not only the compression and posterior bowing of the lamina cribrosa but also the posterior migration of the laminar insertion, which is attributable to physical disruption or remodeling or both [10], is involved in glaucomatous cupping. The advent of enhanced depth imaging spectral-domain optical coherence tomography (SD-OCT) has made it possible to examine the lamina cribrosa in vivo [11]–[15]. Using this technology, Park et al. reported that the anterior laminar insertion (ALI) was displaced more posteriorly in the superior and inferior regions than in the nasal and temporal regions in healthy subjects [16]. However, to the best of our knowledge, lamina insertion has never been evaluated in glaucoma patients.

We hypothesized that posteriorly displaced ALI position is a component of optic nerve head remodeling in glaucoma. Although it was demonstrated in experimental glaucoma model, no study has addressed this issue in human patients. The purpose of the present study was to compare the ALI position of primary open angle glaucoma (POAG) patients and healthy subjects, and to determine factors associated with the more posteriorly located ALI.

## Patients and Methods

This prospective study enrolled newly diagnosed glaucoma patients and age-matched healthy subjects who visited Seoul National University Bundang Hospital between March 2013 and March 2014. Written informed consent was obtained from all subjects. This study was approved by the Seoul National University Bundang Hospital Institutional Review Board and followed the tenets of the Declaration of Helsinki.

All participants underwent comprehensive ophthalmic examinations that included best-corrected visual acuity (BCVA), Goldmann applanation tonometry, refraction tests, slit-lamp biomicroscopy, gonioscopy, dilated stereoscopic examination of the optic disc, disc photographs (EOS D60 digital camera, Canon, Utsunomiyashi, Tochigiken, Japan), central corneal thickness measurement (Orbscan II, Bausch & Lomb Surgical, Rochester, NY, USA), axial length measurement (IOL Master version 5, Carl Zeiss Meditec, Dublin, CA, USA), SD-OCT (Spectralis, Heidelberg Engineering, Heidelberg, Germany), SS-OCT (DRI-OCT1, Topcon, Tokyo, Japan), and standard automated perimetry (Humphrey Field Analyzer II 750, 24–2 Swedish interactive threshold algorithm, Carl Zeiss Meditec).

To be included, eyes had to have a BCVA of 20/40 or better, a spherical equivalent range from −6.0 diopters to +3.0 diopters, cylinder correction within ±3.0 diopters, and no history of intraocular or corneal refractive surgery. The exclusion criteria were [17] a tilted disc (defined by tilt ratio – the ratio between the longest and shortest diameters of the optic disc – over 1.3 [18], torted disc (defined by the torsion angle – the deviation of the long axis of the optic disc from the vertical meridian – over 15° [19], retinal or neurologic diseases that could affect visual function, unreliable visual field tests (fixation loss rate >20%, false-positive or false-negative error rates >25%), and poor-quality SS-OCT images in which peripheral the anterior lamina cribrosa surface could not be imaged at more than 12 meridians. When both eyes were eligible, one eye was randomly chosen for data analysis.

POAG was defined as the presence of an open iridocorneal angle, glaucomatous optic neuropathy with notching, rim thinning, and a retinal nerve fiber layer (RNFL) defect, and corresponding defects in the visual field. Glaucomatous visual field defect was defined as (1) outside normal limits on glaucoma hemifield test; or (2) three abnormal points, with a P<5% probability of being normal and one with P<1% by pattern deviation; or (3) pattern standard deviation of <5% confirmed on two consecutive reliable tests (fixation loss rate ≤20%; false-positive and false-negative error rates ≤25%).

The inclusion criteria for normal subjects were IOP was <21 mmHg, the RNFL thickness as measured by SD-OCT was within the normal range, and normal visual field results. Normal range for SD-OCT was within 95 percentile of the normative database. Normal visual field was the absence of glaucomatous visual field defect and neurological defect.

### Swept-source optical coherence tomography

SS-OCT was performed using the DRI-OCT1 (Topcon, Tokyo, Japan) [20]–[23]. This OCT uses a light source of a wavelength-sweeping laser centered at 1050 nm, with a repetition rate of 100,000 Hz, yielding an 8 µm axial resolution in tissue. The longer wavelength compared with SD-OCT enables deeper posterior penetration [20], which may be advantageous for visualizing the peripheral lamina cribrosa and its insertion site [21]–[23]. The SS-OCT scans were obtained using 6-mm, 12-radial line scans centered on the optic disc. Thirty-two single images were registered and averaged for each line scan. To enhance visibility of peripheral lamina cribrosa, all images were post-processed by adaptive compensation [24], [25], and the measurement was performed by a glaucoma specialist (K.M.L) using Image J (version 1.48, National Insitute of Health, Bethesda, Maryland, USA).

**Figure 1:**
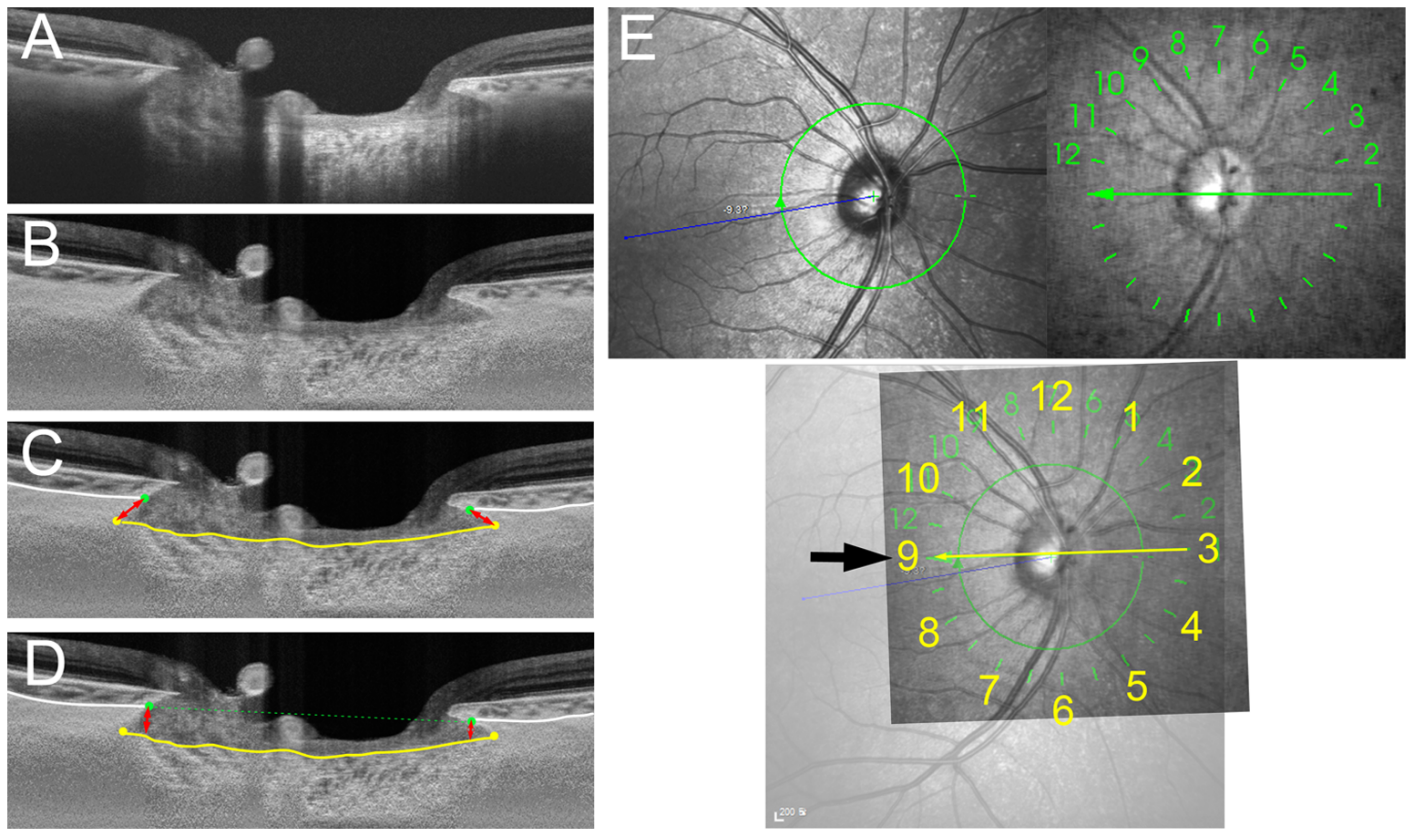
Measurement of the anterior lamina cribrosa insertion (ALI) position and marginal anterior laminar cribrosa depth (mALCSD). **A.** SS-OCT image without labels. **B.** SS-OCT image after adaptive compensation. **C.** Measurement of ALI distance (ALID). The yellow line indicates the anterior lamina cribrosa surface, and the white lines indicate the choroidoscleral interface. ALID (red arrow lines) was defined as the distance from the anterior scleral canal opening (green dots) to the meeting point of the anterior laminar surface and the scleral canal wall (yellow dots). **D.** mALCSD (red arrow lines) was defined as the perpendicular distance from the anterior scleral canal opening plane (green dashed line) to the anterior lamina cribrosa surface (yellow line) at the location of the anterior scleral canal opening (green dots). **E.** Alignment of the SS-OCT scans to the fovea–Bruch's membrane opening center axis (foBMO axis), obtained using Spectralis infrared (IR) fundus photography (upper left). The SS-OCT IR fundus photography (upper right), which contains 12 lines indicating the location of the scan, was overlapped with the Spectralis IR fundus photograph (lower). The SS-OCT radial scan line that was closest to the foBMO axis (thin arrow) was defined as the 9 o′clock meridian (thick arrow). Note that the numbers in the SS-OCT IR fundus photography represent the serial number of the scans, not the clock-hour meridian.

The clock hour location of the medians for the radial scans was determined as the closest meridian to the fovea-Bruch's membrane opening axis (foBMO axis) to be the 9 o′clock position in all patients (Fig. 1) [26]. This was to correct for potential cyclotorsion of the eye or head tilt during image acquisition. As the SS-OCT does not display the foBMO axis, the infrared fundus photograph of SS-OCT (which contains 12 lines indicating the location of the scan) was overlapped with a Spectralis IR (Heidelberg Engineering, Heidelberg, Germany) fundus photograph using commercial software (Photoshop CC, Adobe Systems). With this approach, the maximum degree of inconsistency in regionalization among patients would be 7.5°. The clock hour was assigned based on right-eye orientation.

**Figure 2:**
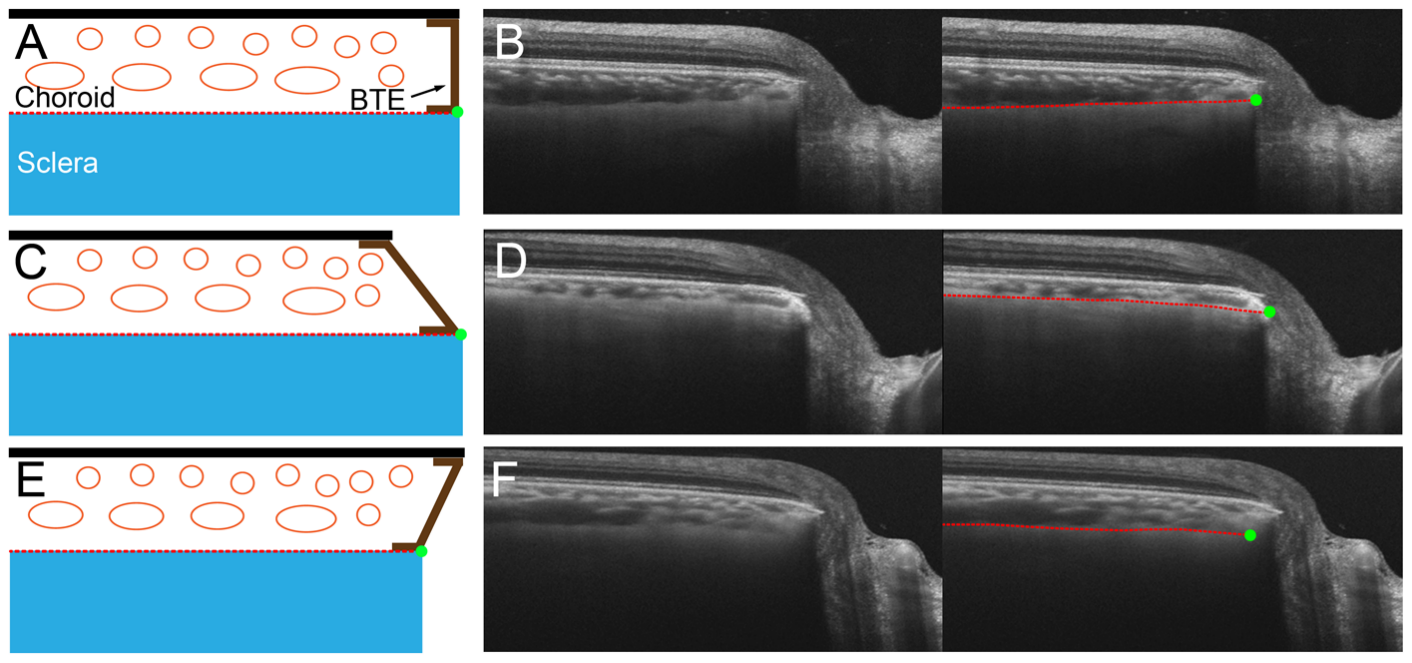
Schematic diagram and sample images for detecting the anterior scleral canal opening (ASCO) in various types of border tissue of Elschnig (BTE). (**A,B**) non-oblique BTE, (**C,D**) externally oblique BTE, (**E,F**) internally oblique BTE. **B, D, F.** Left column is SS-OCT image without label, and right column is SS-OCT image with label. Anterior scleral surface (red dashed lines) is followed to the optic nerve head, and projected to define the ASCO (green dots).

ALI was defined as the intersection of the scleral canal wall or the base of border tissue of Elschnig and the anterior surface of the lamina cribrosa in each of the 12 radial scans. Using these 12 scans, the ALI position was assessed at every- half- clock hour (24 meridians, both sides of each scan) by measuring the following 2 parameters: (1) ALI distance (ALID) – defined as the distance from the anterior scleral canal opening (ASCO) to the ALI; and (2) marginal anterior lamina cribrosa surface depth (mALCSD) – defined as the perpendicular distance from the ASCO plane to the anterior lamina cribrosa surface (Fig. 1). To determine ASCO, the anterior scleral surface plane was traced and/or projected to the optic nerve head (Fig. 2). The ALID and mALCSD were measured by an observer (K.M.L.) who was masked to the clinical information using both original images and those processed by adaptive compensation.

### Data analysis

To measure the interobserver reproducibility of measurement of ALID and mALCSD, 2 observers (K.M.L. and T-W.K.) measured ALID and mALCSD in 21 randomly selected eyes (84 meridians; superior, inferior, nasal, and temporal meridians in each patient), and the intra-class correlation coefficients (ICC) were calculated. Intraobserver reproducibility was assessed based on the 2 measurements by one observer (K.M.L) in the same manner. ALID and mALCSD were compared between the POAG patients and healthy subjects using an independent *t* test. To overcome multiple comparisons of the 24 ALI positions, Bonferroni correction was applied and the cutoff for statistical significance was set at *p*<0.002. Factors associated with ALID and mALCSD were also assessed using multiple linear regression analysis. Statistical analyses were performed with Statistical Package for Social Sciences version 18.0 for Windows (SPSS, Chicago, IL, USA). Except where stated otherwise, the data are presented as mean ± SD values.

## Results

One eye from each of 53 patients with POAG (29 male and 24 female) and one eye from each of 53 age-matched normal subjects (23 male and 30 women) were included in the analysis. Of these, 5 eyes were excluded because the disc was tilted or torted, and 1 was excluded because the peripheral anterior lamina cribrosa could not be visualized in more than half of the meridians, leaving a final sample of 50 glaucoma eyes (22 female) and 50 control eyes (29 female). Comparisons between POAG and age-matched control groups yielded no significant differences in age, BCVA, refractive errors, central corneal thickness, and axial length. The untreated IOP (average of at least two measurements before initiating any IOP-lowering treatment) was higher in the POAG group than in healthy subjects (Table 1).

**Table 1:** Characteristics of the patients with primary open-angle glaucoma (POAG) and the normal healthy (control) subjects.

	POAG (*N* = 50)	Control (*N* = 50)	*p*
Male∶female	28∶22	21∶29	0.230*
Right∶left	24∶26	25∶25	1.000*
Age	60.9±11.2	61.0±11.5	0.972†
logMAR	0.09±0.09	0.11±0.10	0.176†
Refractive errors (D)	−0.07±1.64	0.49±1.88	0.123†
Central corneal thickness (µm)	553±30	558±31	0.438†
Untreated IOP (mmHg)	16.0±3.6	13.5±2.5	<0.001†
IOP scan (mmHg)	12.1±3.5	13.5±2.6	0.023†
Axial length (mm)	23.8±1.2	23.7±1.1	0.794†
Mean deviation	−6.68±6.48	−0.30±1.38	<0.001†

^*^Calculated using the chi-square test.

† Calculated using the independent *t* test.

LogMAR  =  logarithm of the minimum angle of resolution; D =  diopters; IOP  =  intraocular pressure; IOP scan  =  IOP at the time of SS-OCT.

**Figure 3:**
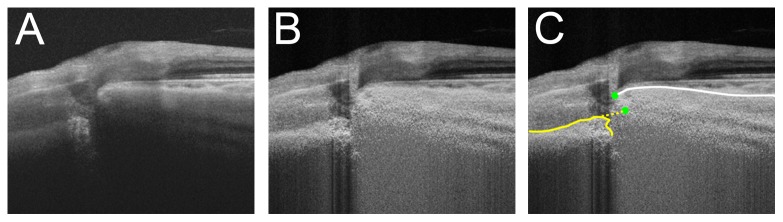
ALID measurement in eyes with an acquired pit. **A.** SS-OCT image without labels. Note that the lamina cribrosa is disinserted from the adjacent sclera. **B.** SS-OCT image after adaptive compensation. **C.** ALI was defined using an imaginary extension line (yellow dashed line) in eyes with a pit.

Table 2 shows the number of eyes for which either ALID or mALCSD was not measurable in each meridian due to invisibility of the ALI or peripheral anterior laminar surface. The ALI position was more often undetectable in the POAG patients than in the healthy subjects. The mALCSD was measurable in nearly all cases in both the POAG and control groups (Table 2). The mALCSD showed good correlation with ALID (Pearson's correlation  = 0.895, *p*<0.001). The ALI position was undetectable in eyes with a deeply located lamina cribrosa, as evidenced by a larger mALCSD for eyes with an undetectable ALI than for those in which the ALI was detectable (327±106 vs 221±79 µm, *p*<0.001). In eyes with an optic disc pit, the ALI was measured from the extension line of the anterior lamina cribrosa surface (Fig. 3). An acquired optic disc pit was observed in nine of the POAG cases.

**Table 2:** Number of cases for which either the anterior lamina cribrosa insertion distance (ALID) or the marginal anterior laminar cribrosa depth (mALCSD) was not measurable in each meridian.

Clock hour	ALID			mALCSD		
	POAG (*N* = 50)	Control (*N* = 50)	*p*	POAG (*N* = 50)	Control (*N* = 50)	*p*
3	12	10	0.629*	2	1	1.000†
4	4	3	1.000†	1	0	1.000†
5	6	4	0.741†	0	1	1.000†
6	3	1	0.617†	0	0	
6.5	0	0		0	0	
7	0	0		0	0	
8	0	0		0	0	
9	0	0		0	0	
10	1	2	1.000†	0	0	
11	5	3	0.715†	0	1	1.000†
11.5	7	3	0.318†	0	0	
12	9	4	0.234†	1	1	1.000†
1	20	15	0.295*	2	1	1.000†
2	17	14	0.517*	1	2	1.000†

^*^ *p* values were calculated by the Chi-square test.

† *p* values were calculated by the Fisher's exact test.

ALID  =  anterior lamina cribrosa insertion distance; mALCSD  =  marginal anterior lamina cribrosa surface depth; POAG  =  primary open angle glaucoma.

The measurement of ALID showed excellent intraobserver reproducibility {ICC (1, 1) for the superior  = 0.995 (0.988–0.998); inferior  = 0.997 (0.992–0.999); nasal  = 0.994 (0.985–0.998); and temporal regions  = 0.992 (0.980–0.997)}, and interobserver reproducibility {ICC (2, 1) for the superior  = 0.993 (0.982–0.997); inferior  = 0.994 (0.985–0.998); nasal  = 0.992 (0.980–0.997); and temporal regions  = 0.992 (0.980–0.997)}. The measurement of mALCSD also yielded excellent intraobserver reproducibility {ICC (1, 1) for the superior  = 0.995 (0.987–0.998); inferior  = 0.987 (0.967–0.995); nasal  = 0.995 (0.987–0.998); and temporal regions  = 0.996 (0.990–0.998)}, and interobserver reproducibility {ICC (2, 1) for the superior  = 0.970 (0.926–0.988); inferior  = 0.990 (0.976–0.996); nasal  = 0.992 (0.979–0.997); and temporal regions  = 0.990 (0.975–0.996), Table 3}.

**Table 3:** Inter and intraobserver reliability of measuring ALID and mALCSD.

ALID	Intraobserver reliability		Interobserver reliability	
Superior	0.995	(0.988–0.998)	0.993	(0.982–0.997)
Inferior	0.997	(0.992–0.999)	0.994	(0.985–0.998)
Nasal	0.994	(0.985–0.998)	0.992	(0.980–0.997)
Temporal	0.992	(0.980–0.997)	0.992	(0.980–0.997)

mALCSD	Intraobserver reliability		Interobserver reliability	
Superior	0.995	(0.987–0.998)	0.970	(0.926–0.988)
Inferior	0.987	(0.967–0.995)	0.990	(0.976–0.996)
Nasal	0.995	(0.987–0.998)	0.992	(0.979–0.997)
Temporal	0.996	(0.990–0.998)	0.990	(0.975–0.996)

ALID  =  anterior lamina cribrosa insertion distance; mALCSD  =  marginal anterior lamina cribrosa surface depth.

### Comparison of ALID and mALCSD

**Figure 4:**
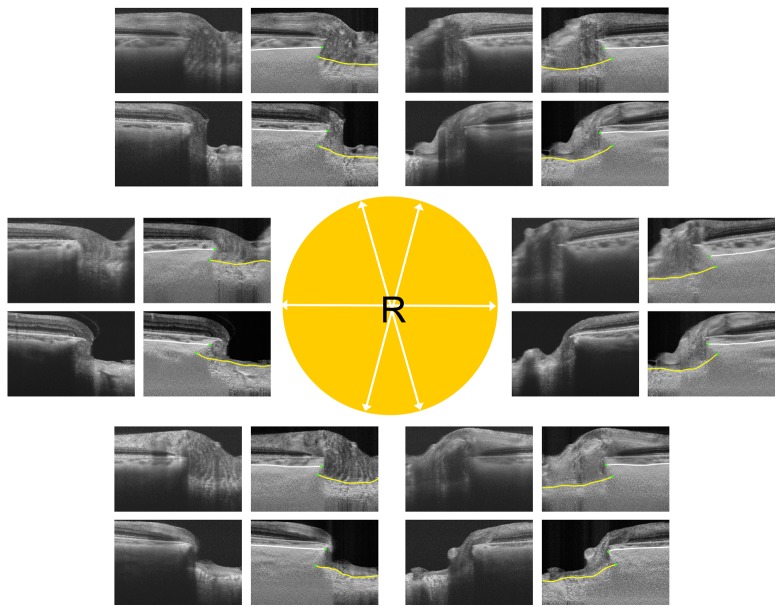
Comparison of ALI positions between eyes from a patient with primary open-angle glaucoma (POAG) and a normal control (both aged 62 years). The images are for the right eye in both patients. White arrows in the central circle indicate the meridians of the scans. The upper and lower images in each pair are from the normal control and POAG eyes, respectively. Yellow lines indicate the lamina cribrosa surface and white lines indicate the choroidoscleral interface. Note that ALID is greater in the POAG eye, with the most prominent differences being observed in the superotemporal and inferotemporal areas.

**Figure 5:**
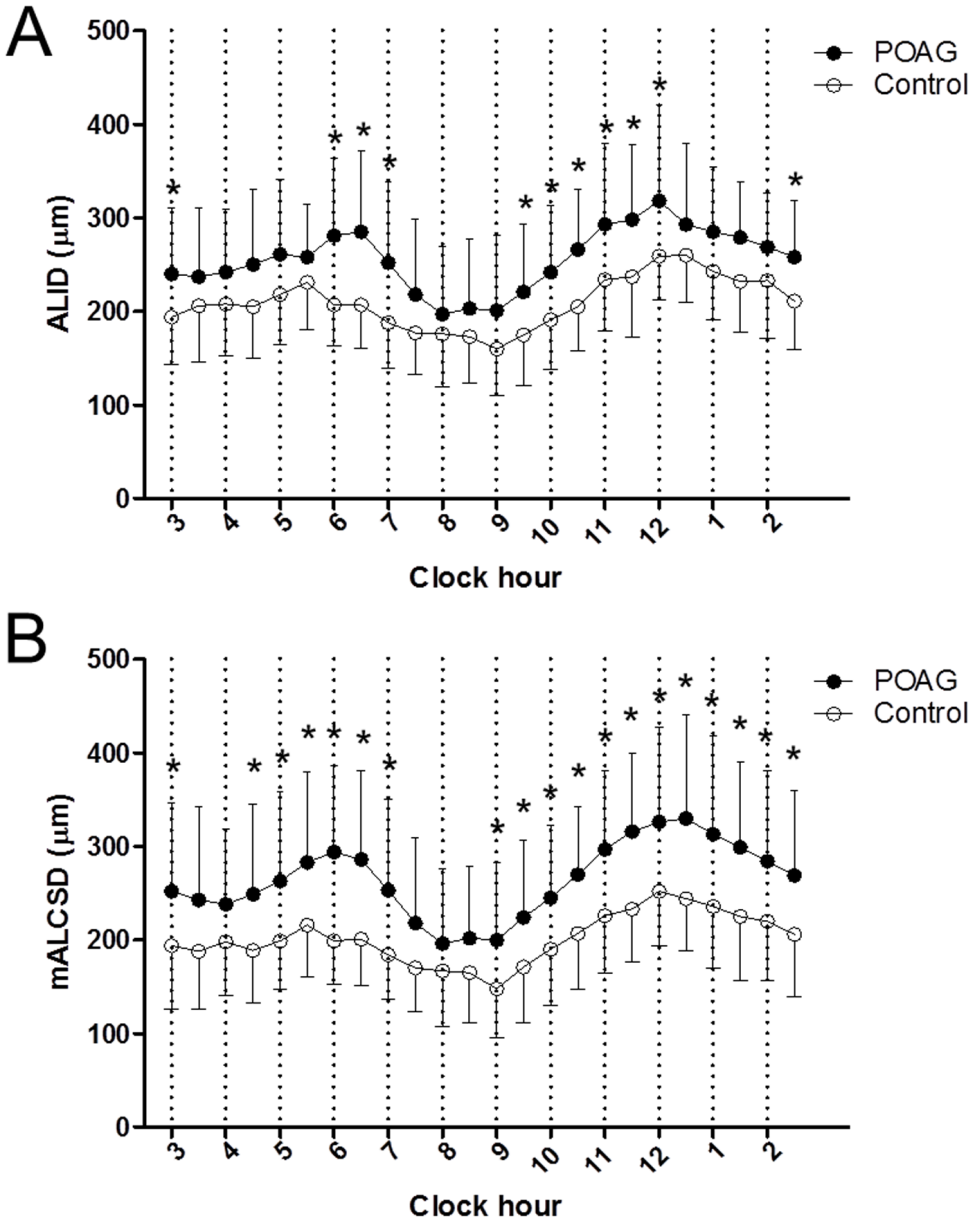
Comparison of ALID (A) and mALCSD (B) between subjects with POAG and healthy controls in each meridian. Significant differences after Bonferroni correction (*p*<0.002) are indicated with asterisks.

ALID was significantly greater in the POAG group than in the normal group (256±54 vs 209±37 µm, *p*<0.001; Table 4, Fig. 4). The greatest difference was observed in the 6.5 o′clock (285±87 vs 207±44 µm, *p*<0.001) and 11.5 o′clock (298±80 vs 237±64 µm, *p*<0.001; Table 4, Fig. 5) meridians. Similarly, mALCSD was significantly greater in the POAG group than in the normal group (232±63 vs 187±40 µm, *p*<0.001; Table 5). The differences were most prominent in the 6 o′clock (294±92 vs 199±47 µm, *p*<0.001) and 11.5 o′clock (316±84 vs 233±57 µm, *p*<0.001; Table 5, Fig. 5) meridians.

**Table 4:** Comparison of ALID between POAG patients and normal healthy (control) subjects.

Clock hour	POAG (µm)	Control (µm)	*p* *
3	240±71	194±51	0.001
4	242±68	208±56	0.009
5	261±80	218±53	0.003
6	281±83	207±44	<0.001
6.5	285±87	207±47	<0.001
7	252±87	188±48	<0.001
8	197±73	176±57	0.102
9	201±81	160±50	0.003
10	242±71	191±53	<0.001
11	293±86	234±55	<0.001
11.5	298±80	237±64	<0.001
12	318±102	259±46	<0.001
1	285±70	243±52	0.008
2	269±58	233±62	0.015
Mean^†^	256±54	209±37	<0.001

^*^ *p* values were calculated by the independent-*t* test. ^†^Mean value was obtained by the average of every half clock hour meridian, although only half of the total measurements were displayed on this table.

ALID  =  anterior lamina cribrosa insertion distance; POAG  =  primary open angle glaucoma.

**Table 5:** Comparison of mALCSD between POAG patients and normal healthy (control) subjects.

Clock hour	POAG (µm)	Control (µm)	*p* *
3	252±94	194±68	0.001
4	238±81	198±57	0.005
5	262±95	199±52	<0.001
6	294±92	199±47	<0.001
6.5	286±95	201±50	<0.001
7	253±98	184±48	<0.001
8	196±80	167±60	0.046
9	200±83	148±53	<0.001
10	245±77	190±60	<0.001
11	297±84	226±61	<0.001
11.5	316±84	233±57	<0.001
12	326±101	252±58	<0.001
1	313±105	236±66	<0.001
2	284±97	220±63	<0.001
Mean^†^	232±63	187±40	<0.001

^*^ *p* values were calculated by the independent-*t* test. ^†^Mean value was obtained by the average of every half clock hour meridian, although only half of the total measurements were displayed on this table.

mALCSD  =  marginal anterior lamina cribrosa surface depth; POAG  =  primary open angle glaucoma.

### Factors associated with ALID and mALCSD

In the healthy subjects, univariate linear regression analysis revealed that a greater ALID was associated with both a higher IOP and younger age. mALCSD was also positively associated with IOP and axial length, and negatively associated with age. In the multivariate analysis, only age was associated with ALID (Table 6). In the POAG patients, untreated IOP and visual field mean deviation showed significant association with ALID and mALCSD. In the multivariate analysis, the visual field mean deviation was associated with both ALID and mALCSD (Table 7).

**Table 6:** Factors affecting ALI in normal healthy subjects.

	ALID				mALCSD			
	Univariate		Multivariate*		Univariate		Multivariate*	
	β	*p*	β	*p*	β	*p*	β	*p*
Age	−1.352	0.003	−1.054	0.050	−1.984	<0.001	−1.706	0.002
IOP	4.590	0.029	2.187	0.328	5.623	0.012	1.812	0.406
CCT	0.098	0.577			0.093	0.620		
SEQ	−2.575	0.376			−3.212	0.294		
AXL	8.093	0.101	1.899	0.711	11.399	0.030	2.332	0.642

ALID  =  anterior lamina cribrosa insertion distance; mALCSD  =  marginal anterior lamina cribrosa surface depth; IOP  =  intraocular pressure; CCT  =  central corneal thickness; SEQ  =  spherical equivalent; AXL  =  axial length.

^*^Variables with P values of less than 0.10 in the univariate regression analysis were included in the multivariate analysis.

**Table 7:** Factors affecting anterior laminar cribrosa insertion in primary open angle glaucoma.

	ALID				mALCSD			
	Univariate		Multivariate*		Univariate		Multivariate*	
	β	*p*	β	*p*	β	*p*	Β	*p*
Age	0.058	0.938			−0.345	0.672		
Untreated IOP	5.102	0.022	1.898	0.389	6.422	0.007	2.905	0.202
CCT	−0.232	0.505			−0.464	0.218		
SEQ	4.657	0.361			1.106	0.843		
AXL	−5.694	0.592			−3.607	0.751		
MD	−4.864	<0.001	−4.366	0.001	−5.581	<0.001	−4.817	0.001

ALID  =  anterior lamina cribrosa insertion distance; mALCSD  =  marginal anterior lamina cribrosa surface depth; IOP  =  intraocular pressure; CCT  =  central corneal thickness; SEQ  =  spherical equivalent; AXL  =  axial length; MD  =  mean deviation.

^*^Variables with P values of less than 0.10 in the univariate regression analysis were included in the multivariate analysis.

## Discussion

This prospective in-vivo case-control study found the ALI to be located more posteriorly in POAG patients than in normal controls in the inferotemporal (6–7 o′clock), superotemporal (9.5–12 o′clock), and nasal (2.5–3 o′clock) meridians (Fig. 5). Similarly, mALCSD was greater in the inferior (4.5–7 o′clock) and superior (9–3 o′clock) meridians (Fig. 5). To the best of our knowledge, this is the first report to compare ALID and mALCSD measured from the anterior scleral opening in POAG patients and healthy subjects.

**Figure 6:**
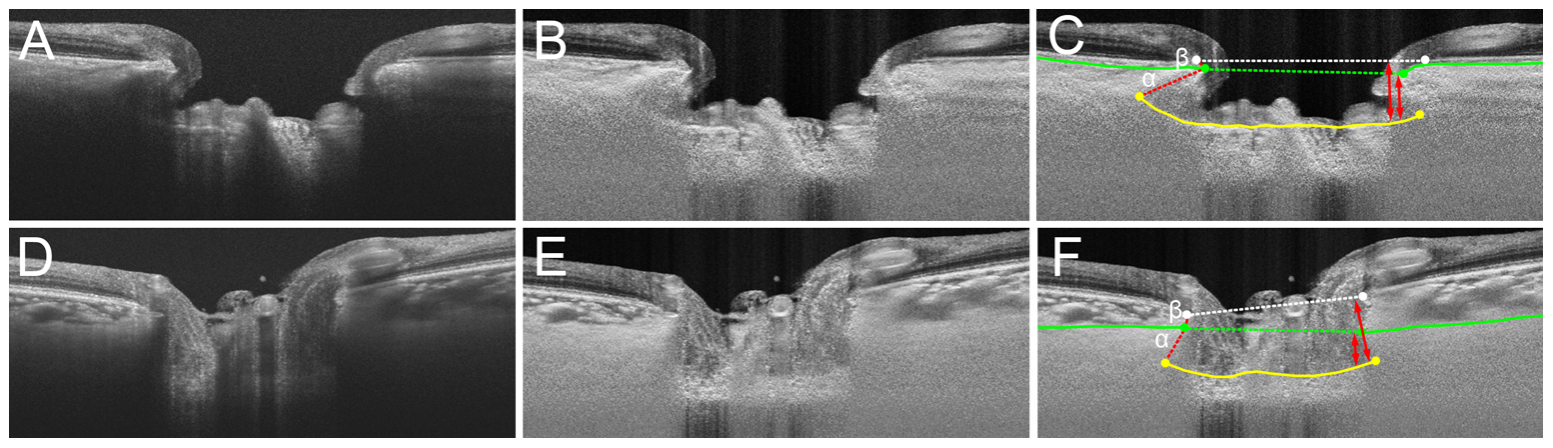
Comparison of ALID and mALCSD based on Bruch's membrane opening (white dashed line) and the anterior scleral canal opening (green dashed line) in a glaucomatous eye (A, B, C) and a normal eye (D, E, F). **A, D.** SS-OCT images without labels. **B, E.** SS-OCT images after adaptive compensation. **C, F.** Yellow lines indicate the lamina cribrosa surface, and green lines indicate the anterior scleral surface. ALID measured from Bruch's membrane opening would be α (red dashed line) +β (red line), while ALID from the anterior scleral canal opening would be α. Note that α+β is similar between **C** and **F**; however, α is noticeably smaller in **F** than in **C**. Similarly, mALCSD is much larger in F when it is measured from the level of BMO (longer double arrows), although the depths from the ASCO (shorter double arrows) are smaller in **F** than in **C**.

Previous studies that measured the anterior laminar surface depth [14], [15] or ALID [16] used Bruch's membrane termination plane as the reference. The current study can be distinguished from those studies because the ALI position was measured using the anterior scleral opening level as the reference. The lamina cribrosa is a collagenous extension of the sclera. It is formed by ingrowth of the surrounding sclera after the optic nerve has already been formed in the fetal period [27], [28]. It is therefore reasonable to measure ALID from the anterior scleral opening and not from the level of Bruch's membrane opening to assess the extent of posterior migration of the ALI. This matter is particularly important because choroidal thickness may vary among patients [29]. In eyes with a thick choroid, ALID would be overestimated even in eyes without any or only a small degree of posterior migration of the ALI. In contrast, ALID would be underestimated in eyes with a thin choroid, even with substantial posterior migration of the ALI (Fig. 6). Use of the anterior scleral opening level as the reference avoids these measurement errors.

In addition to the ALID, the mALCSD was also measured in the present study because the ALID was not always visible. mALCSD is the summation of the vertical component of ALID and the effect of posterior bowing of the lamina cribrosa. However, given the short distance between the ALI and the lamina cribrosa surface where mALCSD was measured, the effect of posterior bowing may be small. Thus, we considered that mALCSD can be used as a surrogate parameter that may indirectly reflect ALID. Since mALCSD is measured perpendiculary from the the anterior sclera edge, it was more frequently measurable than ALID (Table 2). The difference of mALCSD between the POAG patients and healthy subjects was similar to that of ALI in each meridian.

In the healthy subjects, the ALID and mALCSD were negatively correlated with age. We speculate that it could be associated with age-related changes in the connective tissue. Maintaining a high rate of matrix remodeling with matrix metalloproteinase is an important mechanism for conserving the plasticity of the lamina cribrosa in physiologic conditions [30]. Since aging generally involves increased sequestration of matrix metalloproteinase (MMP) and a reduced turnover of the extracellular matrix [31], it can be supposed that older eyes would have a thicker and stiffer lamina cribrosa. Human ex-vivo studies have found that advancing age is associated with a thicker lamina cribrosa [32] and changes in the composition of the extracellular matrix [33], [34]. Being consistent with this, we have reported that the thickness of the central lamina cribrosa increases with aging [35]. This highlights the unique compliance and biomechanics of the aged lamina cribrosa [36], [37]. We speculate that thickening and stiffening of the lamina cribrosa would lead to a smaller ALID and mALCSD. Meanwhile, age was not associated with either ALID or mALCSD in the glaucoma patients. The effect of age might be masked by a more influential factor (e.g., glaucoma severity and IOP-related stress) in them.

In the glaucoma patients, untreated IOP and mean deviation showed a significant association in the univariate analysis. However, only visual field mean deviation remained significant in the multivariate analysis. This finding suggests that the ALI position is not simply dependent on the level of IOP but associated with overall factors involved in the glaucomatous optic nerve damage. In the development of glaucomatous excavation, not only the IOP-related stress itself but also the active remodeling process which is controlled by ECM degrading enzymes (e.g, MMPs) contributed by reactive astrocytes are involved [8], [38]. Such process would continue throughout the disease up to the end stage of disease, thereby leading larger ALID found in eyes with more advanced disease [9]. The most prominent group difference was observed in the inferotemporal (6–7 o′clock) and superotemporal (9.5–12 o′clock) meridians, where glaucomatous damage occurs preferentially. It is generally considered that the lamina cribrosa is less dense superiorly and inferiorly, rendering those areas more susceptible to pressure-induced damage [39]. Our findings suggest that the connection between the lamina cribrosa and the peripapillary sclera is also weaker in these sectors than in the temporal and nasal sectors, thereby rendering them more susceptible to pressure-induced stress. Alternatively, this finding could suggest that tissue remodeling of the lamina cribrosa, which is largely mediated by astrocytes [8], [38], [40], is more vigorous in this region. It is also possible that astrocytes are more strongly activated in this region due to the greater strain on the laminar beams in this area.

In the present study, 92% of the studied POAG patients had an untreated IOP of <21 mmHg over multiple measurements on different days. This suggests that even though their IOP was within the statistically normal range, IOP-induced stress is associated with POAG. Recent computational modeling studies have demonstrated that the mechanical stress imposed on the optic nerve head may vary markedly in eyes with the same IOP, depending on ocular geometry factors such as scleral thickness [41]–[44]. Based on this notion, additional factors may amplify the IOP-derived stress imposed on the optic nerve head in normal-tension glaucoma. Alternatively, Ren et al. reported that the cerebrospinal fluid (CSF) pressure is lower in patients with a normal IOP [45]. Wang et al. subsequently reported that the orbital CSF space was narrower in patients with a normal IOP, consistent with their earlier study [46]. The presence of a low CSF pressure may increase the translaminar pressure gradient, leading the lamina cribrosa to the same condition as in patients with increased IOP and normal CSF pressure.

The existence of posterior migration may have several important implications for the pathogenesis of glaucomatous optic neuropathy [10]. First, posterior migration of the ALI may be considered an additional mechanism of optic-disc cupping. It may be particularly relevant in the presence of peripheral excavation and neural rim loss which are not explained by the posterior migration of the central lamina cribrosa alone [9]. Second, posterior migration of the lamina cribrosa would shorten the distance from the anterior laminar surface to the retrolaminar subarachnoid space. This would result in a steeper translaminar pressure gradient in the peripheral scleral canal, which may increase the impairment of the axoplasmic flow, especially in the peripheral scleral canal [47], [48]. Third, the blood supply to the optic nerve head enters largely from the periphery [5]. Thus, structural alterations in the lamina cribrosa insertion may be accompanied by a change in the vascular supply to the optic nerve. Fourth, disruption or remodeling of the laminar beams may occur during the process of posterior migration, which may in turn result in rupture of the capillaries inside the laminar beams and disc hemorrhage [9], [49]. In line with this concept, we recently demonstrated the occurrence of a structural alteration around the time of disc hemorrhage [49].

This study has limitations. First, the ALI position was not always visible in all meridians. However, it is not likely that this we do not consider that this affected our conclusions. The ALI is likely to be invisible in eyes with a deeply located lamina cribrosa. Consistent with this notion, mALCSD was larger in eyes in which the ALI position was invisible. In addition, the ALI position was more often invisible in the POAG patients. This would lead to underestimation of the average ALID for the entire POAG patients. Thus, any potential bias derived from this limitation would actually reinforce our finding. We consider that the true difference between the POAG patients and healthy subjects is greater than was revealed by the present study. Second, in eyes with an optic disc pit, we measured ALID using an extension line. This measurement is probably inappropriate because the ALI does not exist in such eyes. However, an acquired pit is thought to be a result of optic nerve remodeling associated with lamina cribrosa disinsertion [23], [50], [51]. Postulating that an acquired pit is part of a spectrum of lamina cribrosa displacement, we could not exclude the POAG eyes with pits. Our measurement based on an extension line of the lamina cribrosa implicates the minimum approximation of ALI (i.e., the actual ALID would be the same or larger than our approximation in these eyes). Since acquired pits existed in only POAG patients, with an acquired pit, this limitation also reinforces our finding. Third, we excluded the eyes with tilted or torted optic disc. This was because those eyes might have distorted optic nerve head structure, and thus confounded our analysis. Due to this study design, our data cannot be applied to eyes with tilted or torted optic disc.

In conclusion, the ALI position is located more posteriorly in POAG eyes than in healthy eyes, and this tendency is most prominent in the superotemporal and inferotemporal sectors. The ALI position is correlated with the visual field mean deviation. These findings support the notion that the posteriorly located lamina cribrosa insertion is an important component of glaucomatous optic nerve excavation.
