# Correction: Anterior Lamina Cribrosa Insertion in Primary Open-Angle Glaucoma Patients and Healthy Subjects

**DOI:** 10.1371/journal.pone.0151229

**Published:** 2016-03-03

**Authors:** Kyoung Min Lee, Tae-Woo Kim, Robert N. Weinreb, Eun Ji Lee, Michaël J. A. Girard, Jean Martial Mari

There is an error in the Funding section. The correct funding information is as follows: The work was supported by a grant from the Seoul National University Bundang Hospital Research Fund (02-2014-047), the National Research Foundation of Korea funded by the Korean Government (no. 2013R1A1A1A05004781) and an unrestricted grant from Research to Prevent Blindness (New York, NY). The funding organizations had no role in the design or conduct of this research.
